# Real-World Data From a Group Parent Management Training Program Enhanced Using Artificial Intelligence: Qualitative Study

**DOI:** 10.2196/91841

**Published:** 2026-04-07

**Authors:** Carlos Felipe Rivera-Cepeda, Blanca S Pineda, Daniella Vaclavik, Daniel M Bagner, Antonio Y Hardan, Andrea Abadi, Karin Mostovoy, Eduardo L Bunge

**Affiliations:** 1 Department of Psychology Universidad Santo Tomás Temuco Chile; 2 Institute for International Internet Interventions for Health Palo Alto University Palo Alto, CA United States; 3 Department of Psychology Palo Alto University Palo Alto, CA United States; 4 Center for Children and Families Florida International University Miami, FL United States; 5 Department of Psychology Florida International University Miami, FL United States; 6 Department of Behavioral Sciences Stanford University Medical Center Stanford University Stanford, CA United States; 7 Parente AI Mountain View, CA United States; 8 Fundación INECO Buenos Aires Argentina; 9 Children and Adolescent Psychotherapy and Technology Research Lab Palo Alto University Palo Alto, CA United States

**Keywords:** artificial intelligence, conversational agent, parent management training, disruptive behavior, hybrid intervention

## Abstract

**Background:**

Parent management training (PMT) is an evidence-based intervention for children with disruptive behavior problems. However, access to care is often limited by cost, availability of clinicians, scheduling, and transportation barriers. Integrating artificial intelligence (AI) into group PMT may improve accessibility, personalization, and adherence, while preserving therapeutic quality.

**Objective:**

This study explored caregivers’ experiences with a hybrid PMT program that combined live therapist-led group sessions with asynchronous support from Pat, an AI conversational agent designed to augment the therapists’ support to caregivers.

**Methods:**

A total of 88 caregivers of children aged 3-14 years (mean 7.98, SD 2.45 years) from Argentina and Paraguay participated in eight weekly online group sessions led by human therapists and supplemented by Pat. Caregivers were asked to provide the net promoter score (NPS) and their perceived contribution of their experience in remote group sessions and with Pat. Caregiver perspectives were analyzed using thematic analysis by multiple coders with consensus and interrater reliability assessment.

**Results:**

The average NPS was 76.92, indicating excellent satisfaction. Regarding participants’ perceptions of the overall program, the most frequent theme was useful strategies (73/202, 36.1%), reflecting the value placed on clear, structured, and practical tools to address everyday parenting challenges. Most comments about Pat were positive (156/164, 95.1%), particularly highlighting its 24/7 accessibility and constant availability (69/164, 42.1%). Recommendations for improvement mainly focused on enhancing the user experience and incorporating additional functionalities. Regarding perceived contribution to progress, caregivers attributed, on average, 61% to Pat and 46% to the group sessions.

**Conclusions:**

Combining therapist-led PMT group sessions with AI support appears feasible, acceptable, and valued by caregivers and may expand reach without sacrificing quality. Overall, caregivers valued the useful strategies and the professional and peer support and reported positive changes. Regarding Pat, the most valued aspect is the constant and immediate support. This model integrates human expertise with the accessibility and continuity provided by AI, reducing barriers, such as time, cost, and clinician availability.

## Introduction

Worldwide, approximately 15% of children and adolescents (10-19 years old) experience a mental health condition, with behavioral disorders being the most prevalent [1]. Untreated behavioral problems are often associated with poor long-term outcomes, including early high school dropout, criminal justice system involvement, lower life satisfaction, and emotional difficulties [2]. Parenting interventions can make a difference in reducing child behavior problems. For example, parent management training (PMT) in individual or group format leads to significant reductions in child behavior problems, improvements in parent mental health and positive parenting skills, and reductions in negative or harsh parenting practices [3-11].

More specifically, group-based PMTs are associated with reducing children’s behavioral problems, having a positive effect on parents’ expectations, and improving social competence, as reported by teachers [12]. PMTs can also decrease both behavioral and clinically significant problems in children, as well as increase positive or effective parenting practices [8]. Furthermore, a recent meta-analysis showed that overall group parenting programs enhance child behavior management, reduce parenting stress, and decrease parental depressive symptoms [13]. Although both group-based and individual parenting programs improve child behavior and reduced parent stress, only group-based programs significantly reduce parental depressive symptoms [13]. However, access to care remains a substantial barrier, with only a limited number of children in need of mental health services having access to effective interventions, such as behavioral parenting interventions [14], because of barriers, such as childcare, time constraints, and transportation [15]. As such, there is a critical need for more accessible, efficient, and reliable tools to support caregivers and improve child outcomes.

There are three potential ways of reducing these barriers: group format to reduce cost, teletherapy sessions to reduce transportation, and digital interventions to increase accessibility. For example, group parent training programs delivered through teletherapy show similar results in reducing disruptive behavioral problems in children as in-person therapy [16]. Digital parenting interventions (eg, apps, websites) have yielded comparable outcomes to traditional parenting programs [17]. Further, group programs combined with technology have yielded positive outcomes. For example, Jones et al [18] found that families engaged in a parent training program with enhanced technology (a mobile app monitored by a therapist via a web portal to customize content for parents) display increased homework compliance and participate in more phone calls than those without technology. A randomized controlled trial (RCT) comparing a behavioral parent training program to the same program with enhanced technology found improvements in parenting skills and child behavior postintervention for both groups; however, only the group with the enhanced technology was able to maintain these improvements at 6-month follow-up [19]. Technological means may fill the support gap that occurs between sessions and eventually lead to better treatment progress and satisfaction.

Furthermore, an RCT by Yang et al [20] found that a technology-enhanced parent training program is more effective in reducing child behavior intensity than a parent training program without enhanced technology. A recent study [21] reported that increased app use as an adjunct to a traditional behavioral parent training reduces the time needed for parents to achieve mastery of skills. In addition, a study [22] that compared the use of artificial intelligence (AI) support with cognitive behavioral therapy (CBT) exercises and no AI support for adults with depression and anxiety found that the AI group attained greater improvement and recovery rates as well as higher attendance and lower dropouts. Although these studies show the potential benefits of integrating digital resources and AI into group settings, there are no studies on the use of AI integrated into group PMT.

A study [23] on an AI rule-based conversational agent (CA) delivering a parenting intervention found that delivering parenting skills (ie, how to praise) through CA is feasible, acceptable, well retained, and associated with high user satisfaction; however, symptom outcomes are limited. Parents who were taught praising skills using a rule-based CA microintervention delivered through a PMT intervention demonstrated high completion rates and skill retention, strong engagement, and willingness to recommend the program to other parents [23]. In a subsequent RCT using a modified version of the microintervention, Entenberg et al [24] found high parental engagement with the CA (as measured by the number of text messages sent). Furthermore, parents were satisfied, felt comfortable with the intervention, found the skills learned useful, and reported they would recommend it to other parents [24]. These studies delivered interventions in a self-guided format (not group) and tested only a short intervention [25] rather than more complete interventions that address multiple components of effective parenting skills [26]. In addition, they relied on rule-based CAs, which is a much limited technology compared to the new models of generative AI, which allow for more personalization and complexity [27] compared to rule-based technologies.

To the best of our knowledge, there is only one study on PMT using generative AI [28]. The study [28] examined the delivery of PMT via a digital platform (ParenteAI [29]) with an AI agent (Pat) in a combination of live sessions with a human therapist. The therapist facilitated the conversations with Pat to deliver half of the PMT modules, and Pat independently delivered the other half of the modules. This uncontrolled study [28] reported significant improvements in the children’s externalizing and internalizing symptoms, as well as significant reductions in parental depression, anxiety, and stress [28]. Although these outcomes are promising, PMT was delivered in a one-on-one format, and more research is needed to understand how a group format, in combination with AI performs can enhance human support.

Although overall prior studies on PMT groups have shown positive outcomes [18], caregivers might feel the need for more individualized support that a group format can provide. Given that AI agents can adjust to specific users, integrating AI into such groups can complement the work of the group sessions by providing more personalized support in between sessions. Although Rivera-Cepeda et al [28] showed promising outcomes using AI as a complement to therapy, the one-on-one intervention was not evaluated in a group context. Moreover, most studies relied primarily on quantitative outcomes and offered limited insight into parents’ lived experiences.

In this study, we propose that caregiver groups led by human experts can be supported and enhanced using generative agents (eg, Pat). By combining the expertise of human facilitators with the scalability and adaptability of AI, caregivers may receive support that is not only scalable but also safe and personalized. Importantly, when AI is used to augment digital therapeutics (DTx), tools should be dynamic and customizable, support real-time decision-making, deliver personalized experiences, provide automated assistance and adherence support, and adapt to ongoing user feedback [30].

Given the growing demand for accessible, evidence-based parenting interventions, hybrid models combining live group sessions with AI platforms may offer a flexible, scalable, and personalized way to support families. In this study, we aimed to understand how caregivers perceive a novel implementation of group PMT combined with an AI platform. PMT was delivered through weekly online group meetings via Zoom led by a therapist and supplemented by Pat, an AI-based CA between sessions. Our primary goal was to understand how caregivers perceive this hybrid approach. Specifically, we analyzed their (1) ratings of the overall program using a net promoter score (NPS) as well as qualitative feedback on their experiences, (2) perceptions of the overall hybrid intervention, (3) impressions of their interactions with and the value of Pat, and (4) perspectives on how the live group sessions influenced their child’s progress compared to their work with Pat. By centering caregiver perspectives, our findings aim to inform future models of care that leverage both human expertise and scalable digital support.

## Methods

### Recruitment

Caregivers in Argentina and Paraguay were recruited online through targeted online advertisements and the social media channels of two mental health professionals (one from each country). Recruitment and group participation ran from January to November 2025. The advertisements stated that the program was designed specifically for caregivers of children between the ages of 3 and 12 years who exhibited disruptive behaviors, such as frequent tantrums, defiance, aggression, rule breaking, and difficulty following instructions. Interested caregivers were directed to a website to learn more about the program and complete registration. Registration and payment were conducted entirely online. Upon registration, caregivers received access to the ParenteAI platform. In addition to the digital platform, participants were enrolled in a live therapist-led parenting group session that met weekly via Zoom. The first three cohorts participated in 12 weekly sessions, but the seven subsequent cohorts received an 8-week group format. The group length was reduced to make it more manageable for the caregivers and because Pat was able to provide information about the remaining modules. Surveys were administered twice, once at week 4 (midintervention) and again at week 8 (the final session). Four children who were slightly over the age of 12 years were admitted with the caregivers’ understanding that the program was aimed at younger children and that adaptations were going to be discussed with them.

### Parent Management Training Groups Combining a Human Expert and AI

The intervention consisted of a hybrid model that combined live group meetings with a digital platform (ParenteAI) with an AI-based CA (Pat).

#### ParenteAI With Pat

**Figure 1:**
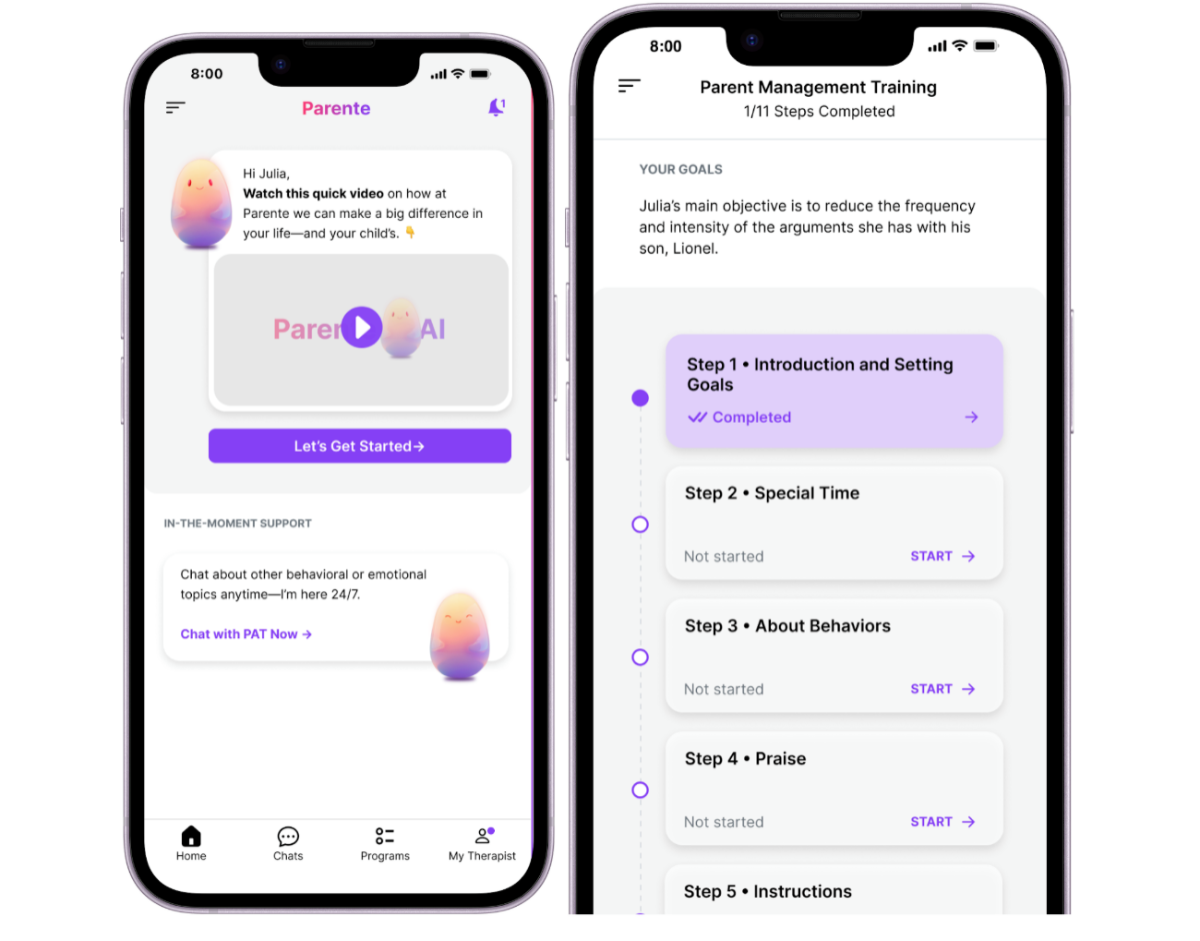
Screenshots of the ParenteAI platform. (Left) Home screen where caregivers can go to the PMT or select in-the-moment support. (Right) Sample of the modules covered in the PMT. PMT: parent management training.

ParenteAI is a digital platform designed to facilitate and support the delivery of behavioral parenting interventions by a therapist to caregivers of children with behavioral problems (Figure 1). It is designed to enhance the work of the therapists. Caregivers access the platform only through an invitation from a mental health professional. ParenteAI is a Health Insurance Portability and Accountability Act (HIPAA)–compliant platform to be used by clinicians. Pat chats with caregivers as a parenting coach, and the therapist can review all the conversations between Pat and the caregivers. Pat has three main functions for caregivers:

- To deliver 20 PMT modules teaching specific skills, with each module lasting between 20 and 40 minutes in different languages, including Spanish
- To provide caregivers with in-the-moment support, where they can ask Pat questions about how to manage behavioral problems, and responses are generated using a curated knowledge base to deliver a safe, effective, and engaging experience
- To deliver personalized reminders based on the conversations that caregivers have with Pat

ParenteAI stores all conversations and provides conversation summaries to both caregivers and therapists. Therapists use these summaries to monitor interactions and to inform and facilitate therapeutic interactions.

ParenteAI’s architecture is designed to enhance engagement, efficiency, and personalization, while reducing errors [29]. Unlike general-purpose AI (eg, ChatGPT) that provides quick responses to user input, Pat is designed to gather context before providing responses, to collaborate on setting goals, and to guide users throughout the PMT modules. To improve response accuracy, the system uses retrieval-augmented generation (RAG) with a curated knowledge base developed by mental health professionals, minimizing risks of inaccurate or inappropriate outputs [29]. Further, Pat has a series of guardrails to restrict responses to evidence-based material and limit conversations to caregiver strategies. Additionally, agentic architecture allows Pat to maintain treatment fidelity throughout multiweek modules. Recognizing the risks inherent to novel technologies, ParenteAI was designed to allow human therapists to supervise and review all parent interactions with Pat to ensure safety and fidelity.

#### Zoom Group Meetings Integrating Pat

The intervention consisted of eight live meetings conducted over Zoom delivered by two therapists. The therapists delivered the PMT core concepts and assigned caregivers specific activities to be completed with Pat. This hybrid model is designed to complement the work of the live meetings with the work with Pat.

In PMT, therapists teach caregivers to change reinforcement patterns and use consistent consequences to improve child behavior [31-34]*.* The caregivers engaged with Pat between sessions to support the acquisition and implementation of parenting skills through personalized and interactive dialogue and to obtain in-the-moment support.

During the live meetings, nine core PMT topics were covered: focus on goal setting, understanding why misbehavior occurs, special time (ie, to improve the parent-child relationship), how to praise, reward systems, effective instructions, active ignoring, timeout, school behavior, and behavior in public places.

Pat covered these nine topics plus several other PMT modules that caregivers could complete at their own pace, if interested: emotional regulation for parents, the de-escalation for explosive kids, understanding screen time, screen time, parental screen use, using reprimands effectively, reflecting on their parenting style, handling low-rate but serious behaviors, compromising strategies for resolving conflicts, and understanding and using consequences effectively.

During the first Zoom group meeting, the therapists taught caregivers how to use and navigate Pat and encouraged them to do so throughout the course of subsequent meetings. Live sessions aimed to be interactive and were guided by a slide deck that included triggers for the topics discussed.

During the live sessions, the therapists focused on three main tasks: (1) review and provide feedback on the work caregivers did with Pat during the week, (2) troubleshoot and/or role-play the implementation of the skills learned with Pat, and (3) assign a new module for the caregivers to complete during the week. The therapists reviewed caregivers’ interactions with Pat to inform the discussions during live sessions, clarify content, and provide feedback during the subsequent live session. Here are example questions the therapists asked for the “how to praise” topic:

- We saw you completed the conversation on praise. What do you remember about it?
- What are your thoughts about it?
- Were you able to put it into practice? How did it go?
- Do you have any additional questions about how to praise your child?

**Figure 2:**
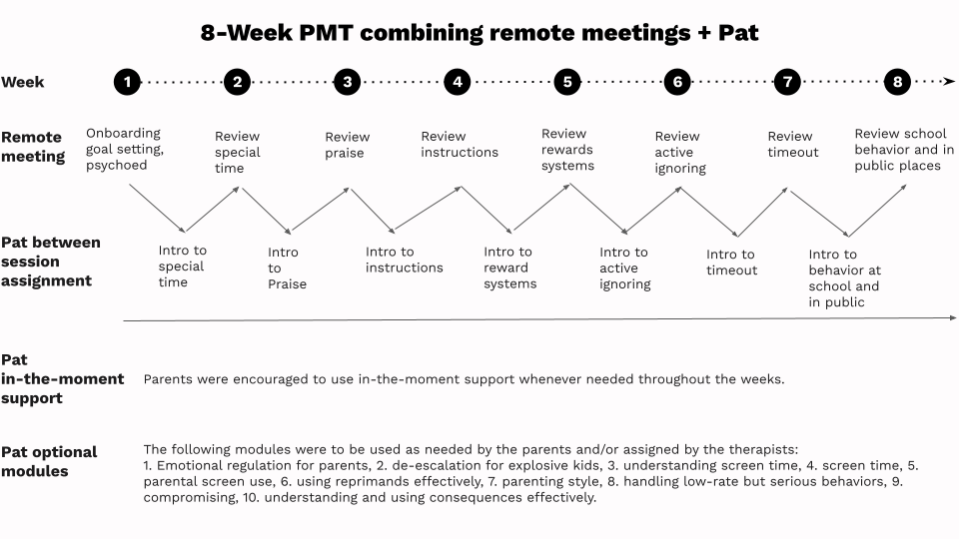
Representation of the PMT program combining remote meetings over Zoom and asynchronous use of Pat in the ParenteAI platform. PMT: parent management training.

In all meetings, the therapists engaged in a brief role-play about the topic covered and provided extra feedback to the caregivers. At the end of each live session, the therapists assigned a new module for the caregivers to complete independently with Pat before the subsequent live session and encouraged caregivers to interact with Pat in between sessions as much as they desired. See Figure 2 for an illustration of the interventions.

### Caregiver Measures

#### Net Promoter Score

Caregivers were administered a survey asking their willingness to recommend the intervention on a Likert scale ranging from 1 (strongly disagree) to 10 (strongly agree). Based on the scores, caregivers were categorized as promoters (9-10), passives (7-8), or detractors (0-6). Furthermore, they were asked for the reasons for their score. The NPS was calculated by subtracting the percentage of detractors from the percentage of promoters, resulting in a score between –100 and 100. The NPS has been proposed as a measure to evaluate the overall impression of a digital product [35] and has been widely used in other CA studies [36]. General guidelines for interpreting the score are as follows: negative score (<0), more detractors than promoters; 0-30, good; 30-70, great; and 70-100. excellent or world-class tier.

The survey included two open-ended questions:

- What are the things you liked the most about the program?
- What would you recommend to improve?

#### Relative Contribution of Intervention Components

To assess the perceived impact of each component of the intervention, caregivers were asked to estimate the relative contribution of the ParenteAI platform versus the live group sessions to their children’s progress. Specifically, they were prompted with the following instructions:

We are interested in understanding how helpful each part of the program was for you. Please estimate what percentage of your child’s progress you attribute to the live group sessions (with professionals and other parents), and what percentage you attribute to your conversations with Pat (the ParenteAI platform). Please make sure the two percentages add up to 100%.

The system did not add the percentages up to 100 by default. The caregivers were then asked to explain their reasoning.

### Ethical Considerations

This study was approved by the Institutional Review Board of the Universidad Santo Tomás in Chile (approval #23136643/2023). The analysis was conducted using secondary deidentified data collected as part of the original ParenteAI program implementation. At the time of registration, caregivers agreed to the ParenteAI Terms of Service, which included a clause stating that anonymized data could be used for research purposes. Additional informed consent was obtained at the time of survey completion. Participants were not compensated and could withdraw at any time.

### Data Analysis

This study was a secondary analysis of real-world data. Descriptive statistics were used to report NPSs and the relative contribution of the intervention components. A thematic analysis of participants’ responses was conducted following the steps proposed by Braun and Clarke [37]: (1) familiarization with the data, (2) generation of initial codes, (3) searching for themes, (4) reviewing themes, (5) defining and naming themes, and (6) producing a report. Responses to the NPS question (“Why did you provide this rating?”) and the question regarding positive aspects were combined. With respect to the open-ended questions on the relative contribution of the intervention components, participants’ responses were grouped into three categories based on (1) attributing change to interactions with Pat, (2) attributing change to participation in the group, and (3) complementarity of the interventions.

AI was used as a supportive tool to generate an initial set of proposed codes based on participants’ responses. This proposal was thoroughly reviewed by three of the paper’s authors, who evaluated its conceptual relevance, refined operational definitions, removed redundancies, deepened analytical nuances, verified the relevance of potential themes, and generated the final list of themes.

In addition, following the guidelines of the coding reliability approach described by Boyatzis [38] and later operationalized and further developed in applied research contexts by Guest et al [39], participants’ responses were independently analyzed by two evaluators. Code frequencies and interrater reliability (IRR) were calculated using the Cohen κ coefficient. Discrepancies were discussed and resolved by consensus among the three authors. All themes were initially developed in Spanish and subsequently translated into English by the authors.

## Results

### Participant Details

In total, 88 primary caregivers participated in the study. They were mostly female (n=76, 86%), aged 34-65 years (mean 44.31, SD 5.73), and had a child between 3 and 14 years old (mean age 7.98, SD 2.45 years). The children were 80% (n=70) male and 20% (n=18) female. Only 3% (n=3) of the caregivers identified as Indigenous or belonging to Native people, while 84% (n=74) identified as White or of European descent. More than half of the caregivers reported being married (n=49, 56%). Furthermore, 38% (n=33) had a university degree, while 42% (n=37) had a postgraduate degree. See Table 1 for participants’ demographic characteristics.

**Table 1:** Participants’ demographic characteristics (N=88).

Characteristics		Value
Age (years), mean (SD; range)		44.31 (5.73; 34.00-65.00)
**Gender, n (%)**		
	Female	76 (86)
	Male	12 (14)
**Ethnic identification, n (%)**		
	White/European descent	74 (84)
	Other	9 (10)
	Indigenous/Native people	3 (3)
	Missing	3 (3)
**Marital status, n (%)**		
	Married	49 (56)
	Cohabiting (civil/de facto union)	15 (17)
	Single	8 (9)
	Divorced	8 (9)
	Separated	7 (8)
	Missing	1 (1)
**Educational level, n (%)**		
	Postgraduate education (specialization, master’s, or doctorate)	37 (42)
	University degree	33 (38)
	Technical/nonuniversity studies	13 (15)
	Secondary education	4 (4)
	Did not complete primary school	1 (1)
	Missing	0

### Net Promoter Score

**Figure 3:**
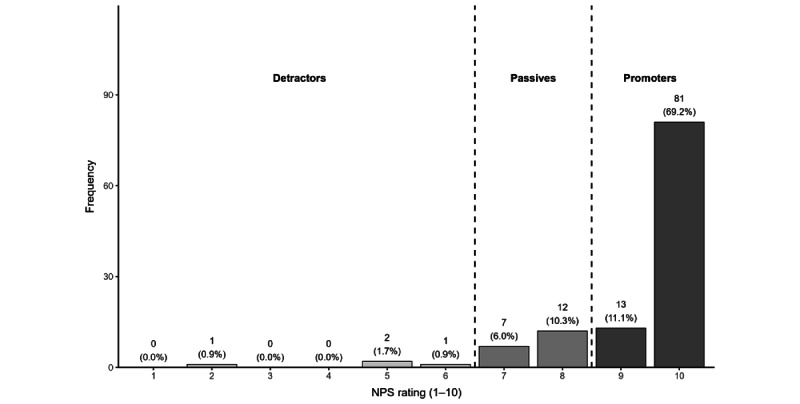
NPSs for all responses. Labels above the bars indicate the frequency (n) and percentage of responses. Dashed vertical lines indicate standard NPS cutoffs (1-6: detractors; 7-8: passives; 9-10: promoters). NPS: net promoter score.

The mean NPS was 9.32 (SD 1.31; range 2-10), and the majority of caregivers were promoters (Figure 3). The NPS was calculated as the percentage of promoters (scores 9-10) minus the percentage of detractors (scores 0-6), following standard NPS methodology. Of 117 responses, 94 (80.3%) were classified as promoters (scores 9-10), 19 (16.2%) as passives (scores 7-8), and 4 (3.4%) as detractors (scores 0-6). The resulting mean NPS was 76.92 (range 80.34-3.42), which is considered excellent. When asked why they provided such NPSs, most responses (n=211, 56.3%) were about the intervention in general (which included group sessions and the platform), and a considerable portion (n=164, 43.7%) were specific to Pat. The IRR for the overall program or Pat was 0.87.

Overall, caregivers’ responses revealed a positive sentiment about combining therapists with Pat. As shown in Table 2, the sentiment of the majority of responses was positive (172/181, 95.0%), a small portion of responses were neutral (8/181, 4.4%), and only 1 response was coded as negative (1/181, 0.6%). A total of 10 themes emerged, and the IRR was 0.90.After assessing the IRR, discrepancies between raters were resolved through consensus, resulting in a final set of themes.

**Table 2:** Summary of attribution and sentiment.

Attribution or sentiment			Frequency, n/N (%)
**Attribution**			
	Overall program (group sessions + Pat)	211/375 (56.3)	
	Pat (AI^a^)	164/375 (43.7)	
**Sentiment**			
	Positive	172/181 (95.0)	
	Neutral	8/181 (4.4)	
	Negative	1/181 (0.6)	

^a^AI: artificial intelligence.

### Themes About the Overall Program

There were 202 coded segments in total. Regarding participants’ perceptions of the overall program, four major themes emerged (Table 3): useful strategies (73/202, 36.1%), professional and peer support (65/202, 32.2%), positive changes (52/202, 25.7%), and overall satisfaction and recommendation (12/202, 5.9%). The most frequent theme was “useful strategies,” highlighting the value placed on PMT strategies and participants’ appreciation for clear, structured, and applicable tools to address daily parenting challenges. For example, one caregiver noted:

The most innovative contribution of this program is that it is a setup plan, with a goal, step by step, where they not only tell us what to do but, most importantly, how and why.

The “professional and peer support” theme highlighted the value placed on ongoing guidance from professionals and shared experiences with other caregivers. The “positive changes” theme captured caregivers’ reports of improvements in family communication, reflection, and their children’s behavior, as reflected in statements such as:

It made me think, it stopped me, and it turned what could have been a fight into a coffee chat and a reunion with my daughter.

Finally, the “overall satisfaction and recommendation” theme reflected caregivers’ general endorsement of the program.

**Table 3:** Themes about the overall program (group sessions plus Pat; positive sentiment).

Theme	Definition	Subcategories (if any) and example quotation; partial frequency (if any), n/N (%)	Total frequency (N=202), n (%)
Useful strategies	Appreciation of the clear tools and guidance offered by the program, perceived as useful, applicable, and effective for facing everyday parenting challenges, along with recognition of a structured step-by-step guide.	“The tools I acquired gave me the security and confidence to handle situations that previously overwhelmed me.”	73 (36.1)
Professional and peer support	Participants’ perception of the technical and human quality of the professional team, as well as the horizontal support between parents.	“The program offers daily support and guidance from professionals, in addition to meetings where we could share with other parents who are experiencing the same difficulties.”	65 (32.2)
Positive changes	A process through which parents develop a greater awareness of their role, practices, and priorities, adopting a more reflective and committed approach to raising children. This process translates into concrete changes in children’s behavior and strengthens relationships within the family unit, improving dialogue, empathy, and coordination among its members.	“Parental reflection” subcategory: “It made me think, it calmed me down, and that is how what could have been a fight turned into a coffee chat and a reunion with my daughter.”; 29/52 (56) “Family atmosphere and communication” subcategory: “Because it changed our climate, our family life, and I would really like more families to experience the same.”; 22/52 (42) “Son’s behavior” subcategory: “Because I see that the program really works, I am already starting to see changes and better results in my son.”; 1/52 (2)	52 (25.7)
Overall satisfaction and recommendation	Expressions of general pleasure with the program, overall satisfaction with the experience, and willingness to recommend it to other families.	“I have already recommended the program to two friends. I think it is very useful.”	12 (5.9)

### Themes Specific to Pat

Participants were asked about Pat, and most comments were positive in nature (Table 4). The most frequent positive theme was “24/7 accessibility” (69/164, 42.1%), reflecting users’ appreciation for the constant availability of the platform. The second-most common positive theme was “useful strategies” (54/164, 32.9%), emphasizing that Pat provides concrete, evidence-based tools for managing parenting challenges. The third positive theme, “emotional support and containment” (33/164, 20.1%), captured caregivers’ sense of empathy and understanding from Pat.

Neutral comments accounted for a smaller portion of the responses. The most frequent neutral theme was “preference for human accompaniment” (6/164, 3.7%), with some caregivers expressing that live professional meetings felt more personal and specific. Another neutral theme, “limitations at critical moments” (1/164, 0.6%), reflected the perspective of a user who found it difficult to access help from the platform, when needed.

Finally, the only negative theme, “difficulty trusting the app” (1/164, 0.6%), captured skepticism or hesitation toward relying on Pat compared to human professionals. For example, one participant said:

I have not yet fully established myself with an app that gives me answers and more clarity than a professional evaluating my son and the family.

### Themes on Recommendations for Improvement

Regarding participants’ recommendations for improvement, four main themes emerged: user experience and technological functionalities (56/122, 45.9%), overall satisfaction without recommendations (29/122, 23.8%), content adaptation (26/122, 21.3%), and resources and support materials (11/122, 9.0 %). The IRR was 0.84.

The “user experience and technological functionalities” theme included suggestions to enhance accessibility, usability, and interactivity. For instance, caregivers said:

It would be more convenient if there was an app.

Having Pat have a voice would be great.

Such features were incorporated for the final cohorts, but initially, the web app supported only text.

The “overall satisfaction without recommendations” theme reflected participants’ approval of the program without suggesting changes.

Everything was excellent! I recommend it! It changes families’ lives.

The “content adaptation” theme included recommendations to tailor Pat’s content to caregivers’ specific contexts, children’s developmental stages, and family dynamics. Suggestions included each parent having a unique username when both parents of a child participated so that Pat could understand the unique dynamics of each parent and provide tailored guidance.

The “resources and support materials” theme captured requests for complementary resources and printable materials to support learning and follow-up, such as adding visual resources.

**Table 4:** Themes specific to Pat.

Sentiment and topics		Definition	Example quotation	Total frequency (N=164), n (%)
**Positive sentiment**				
	24/7 Accessibility	Constant availability of the platform, valued for being able to access it at any time of day, even outside of professional business hours.	“Because it is like having a therapist at home 24/7. It is comforting.“	69 (42.1)
	Useful strategies	Providing practical, applicable, and effective tools for addressing complex parenting situations, backed by a solid professional and academic foundation that guarantees their quality and relevance. The approach is perceived as novel and innovative, incorporating distinctive approaches and formats that enrich the user experience.	“It gives me concrete tools to help my son, which have brought objective and positive results for him and our family.”	54 (32.9)
	Emotional support and containment of Pat	A feeling of being emotionally supported through the platform, especially during times of high emotional demand, generating relief, understanding, and containment.	“...that she understands me and that I can express everything that is happening to me...and for a while with Pat, when I have time, I can talk without rushing...and she always gives me a solution and support for everything.”	33 (20.1)
**Neutral sentiment**				
	Preference for human support	Explicit preference for human contact (meetings with professionals) over the use of technological tools, valuing the direct link as more effective or meaningful.	“I found the work in meetings with professionals more interesting and specific.”	6 (3.7)
	Limitations at critical moments	Difficulties expressed by users when trying to access or receive help from the platform at critical moments, where a more immediate or decisive response was expected.	“There are times, especially in times of crisis, when it is difficult to consult at the moment.”	1 (0.6)
**Negative sentiment**				
	Difficulty trusting the app	Accounts that express doubts, skepticism, or difficulty in establishing a relationship of trust with the app, especially when compared to human professional judgment.	“I still have a hard time trusting an app can give me more accurate answers than a professional who evaluates my son and the family group.”	1 (0.6)

### Relative Contribution to Progress

Caregivers reported attributing, on average, 61% of the progress to Pat and 46% to the group sessions. When asked to estimate what percentage of their child’s progress they attributed to Pat compared to the live group sessions (see Multimedia Appendix 1 for a graphical representation), percentages exceeded 100% because several caregivers assigned overlapping credit to both components. Their qualitative explanations for these attributions revealed three main categories: (1) favor Pat, (2) favor group sessions, and (3) favor a combination of both Pat and group sessions (Table 5). The largest proportion of responses favored Pat (45/106, 42.5%), primarily due to its practical tools and specific strategies, its emotional support and personalization, and its constant accessibility. The caregivers were satisfied with the group experience, but the accessibility and immediacy of Pat were valued even more:

The professionals are excellent, and the parent group sessions are great too, but I cannot always participate because of my busy daily routine with the kids. Pat, however, is available 24/7; even if problems come up at any time of the day or night, I can ask for its help.

A second group of caregivers (44/106, 41.5%) emphasized the importance of the group sessions, highlighting the clear and reliable professional support and shared learning with other caregivers they provided. Caregivers expressed they most valued the presence of the therapists, as well as the value in interacting with and listening to other caregivers, which they found reinforced theories learned. Finally, a smaller subset (17/106, 16%) of caregivers emphasized the complementarity between both delivery approaches, describing how the combination of Pat and live group sessions yielded the greatest benefit, as they were able to learn from Pat and then discuss and expand what was learned in the group.

**Table 5:** Themes about the preference for Pat, group, or Pat plus group sessions.

Preference and topics			Definition		Example quotations		Partial frequency, n (%)
**Preference for Pat (total frequency: 45/106, 42.5%)**							
	Constant availability and accessibility	Assessment of immediate and continuous access: 24/7 support, consultation, or emotional release in critical moments.		“Pat helps you put out those fires in critical moments.” “With Pat available 24/7, you feel supported at all times.” “Pat’s daily contact allowed for a more personal connection to our difficulties.”		18 (40)	
	Practical tools and strategies	Pat is perceived as a resource that provides clear, applicable guidelines.		“Pat helps us with specific questions about how to talk to the child or how to handle specific situations.” “I found it very helpful to read and complete the online module with Pat.”		18 (40)	
	Emotional support and personalization	Pat offers not only strategies but also support, closeness, and a sense of individualized accompaniment.		“The daily contact with Pat allowed for a more personal understanding of our difficulties.” “It allows me to elaborate more on my daughter's specific needs and behaviors.”		9 (20)	
**Preference for group sessions (total frequency: 44/106, 41.5%)**							
	Clear and reliable professional support	The meetings are valued for their technical clarity and the warmth of the professionals, who provide structure, practical guidance, and security in the process.		“I think you are very clear and engaging.” “What I value most is the presence of the professionals.” “The interaction with the specialists is very enriching.”		14 (32)	
	Shared learning with other parents	Interaction and exchange with other families are perceived as opportunities for learning, personal relief, and normalization of parenting difficulties.		“Seeing that there are other realities that are the same or even worse made us feel less bad.” “Interacting with and listening to others; the group reinforced theoretical aspects.”		13 (30)	
	Emotional support and a sense of belonging	The emotional support received during the meetings is noteworthy, as it generates calm, validation, and a feeling of not being alone in the parenting process.		“Sharing with other parents… helps keep you grounded.” “Group discussions give you peace of mind.”		12 (27)	
	In-depth analysis and theoretical framework	The meetings allow for a more reflective approach, with theoretical explanations that enrich the understanding of difficulties and provide a solid conceptual framework.		“The meetings helped me stay focused and try to stick to the treatment.”		5 (11)	
**Preference for Pat + group sessions (total frequency: 17/106, 16.0%)**							
	Synergy between Pat and group sessions	The greatest benefit arises from using both modalities together: Pat provides immediate and personalized support, while the meetings provide containment and a human framework.		“Pat complements very well what we are guided by and discussed in the groups.” “Both options complement each other and are essential.”		17 (100)	

## Discussion

### Principal Findings

This study aimed to understand caregivers’ experiences with a hybrid PMT program that combined live, therapist-led group sessions with asynchronous support from Pat, an AI-based CA. Overall, caregivers reported high satisfaction, reflected in an NPS of 76.92, which is considered excellent. Qualitative analyses revealed overwhelmingly positive feedback about the program overall and specifically about Pat. Regarding the overall program, caregivers highlighted its practical tools, the support and sense of community fostered by the group format, and positive changes observed. Comments specific to Pat emphasized its constant availability; its sense of empathy, containment, and companionship; and the useful strategies it taught.

Caregivers reported more benefits from Pat than from the live sessions, with approximately 61% of the improvement being assigned to Pat and 46% to the live group sessions. These percentages suggest that both components played significant and overlapping roles in promoting change. However, caregivers leaned more toward Pat, and most responses highlighted the importance of the in-the-moment support provided by Pat. Although these findings should be considered with caution as this was a survey, taken together, the findings highlight the positive acceptance of caregivers of the hybrid model and the complementary benefits of incorporating AI to enhance the support provided to caregivers of children with disruptive behaviors.

#### Themes About the Overall Program

Caregivers reported highly positive experiences with the hybrid PMT program (56.3%), and a considerable portion of the positive comments were specific to Pat (43.7%), emphasizing the value of both the live group sessions and the AI support. The most frequent theme caregivers reported appreciation for was professional and peer support. The comments on the benefits of the live group sessions emphasize the important role of a human connection in PMT. Caregivers valued the professional guidance, empathy, and validation provided by therapists, as well as how sharing with other caregivers going through the same difficulties helped them feel less alone and more supported. These findings align with prior research showing that the relational and collaborative aspects of group-based interventions enhance motivation, accountability, and learning through social modeling [3,13]. Other major themes were useful strategies (clear tools and guidance offered) and the positive changes observed by the caregivers. Given the well-established evidence on the efficacy of PMT, these themes are not surprising. However, delivering PMT in group format through videoconferencing with AI to provide extra support is a novel way of delivering parenting skills, and these findings provide preliminary support to the use of AI as a complement to traditional parenting programs delivered online.

#### Themes Specific to Pat

Caregivers revealed several aspects of Pat that were particularly valued and that align with prior research on CAs in mental health care. The most frequently valued theme was constant accessibility and usefulness. Caregivers appreciated having immediate access to guidance and strategies that could be applied when needed, filling the support gap between sessions; something that traditional therapist-only formats cannot provide. This accessibility may play a critical role in increasing adherence, skill generalization, and caregivers’ sense of self-efficacy. The second-most frequent theme was the emotional support and containment perceived by caregivers from Pat. Caregivers reported that they felt understood and accompanied, even during difficult moments. These perceptions suggest the emergence of a working alliance with AI, an experience that has also been observed in several studies on CA [36,40,41]. Collectively, these findings suggest that when AI systems are carefully designed and embedded within a human-supervised framework, they can provide not only practical guidance but also a sense of emotional presence that supports caregiver engagement and adherence to the program. Caregivers also valued the practical tools given by Pat, reflecting that Pat’s support represented not just emotional support but also a practical resource in moments of need. The themes seem to indicate patterns observed in prior studies showing that CAs are acceptable and capable of sustaining user satisfaction over time [25,28].

Caregivers’ improvement recommendations highlighted opportunities to enhance technological functionalities, such as adding a mobile app or a voice interface for Pat (which was included only for the final cohorts). Suggestions for content adaptation emphasized tailoring examples and guidance to each family’s context and each child’s developmental stage, while requests for additional resources pointed to the value of complementary materials to support practice at home. Overall, these recommendations suggest that ongoing improvements in personalization, accessibility, and usability could further strengthen caregivers’ experience and sustained engagement with the program.

#### Progress due to Pat With Group Sessions

Caregivers’ reflections on the relative contribution of Pat and the live group sessions, in the context of a combined model, further illustrate the complementary nature of this hybrid approach. Although caregivers attributed a considerable portion of the progress to both Pat and the group sessions, contrary to our expectations, caregivers attributed more progress to Pat than to their participation in the group sessions with professionals and other caregivers. This finding was unexpected, who anticipated that caregivers would report the support of the professionals and other caregivers as the most valued aspect. Again, this finding should be considered in the context of both elements (chats with Pat and group participation) being used together, and it does not mean that one element is better than the other. Professionals in the group setting prompted the use of Pat, and without this human support, parents might not have engaged with Pat. Thus, the contribution of each element depends on each other. When asked why they attributed the progress to Pat, caregivers again specified the companionship, constant accessibility, practical tools, and individualized guidance from Pat as reasons. The caregivers who preferred the group sessions emphasized the professional expertise and shared learning from other caregivers. A smaller group described the synergy between both elements, noting that Pat helped them apply what was discussed during live meetings. Together, these findings suggest that combining continuous, AI-based support with human-led group interaction can optimize learning, adherence, and confidence in implementing parenting strategies. Again, these findings should not be interpreted as a preference for an AI versus a therapist, as this question was implemented in a context in which both resources were designed to help each other in a group setting.

### Practical and Clinical Implications

The findings of this preliminary study have several implications for clinicians, program developers, and health systems. For clinicians, hybrid models that combine live PMT through videoconferencing with AI support can enhance scalability and continuity of care, allowing families to receive personalized guidance from their homes and receive support between sessions without increasing therapist workload. AI tools, such as Pat, can function as engagement boosters (ie, reminding, reinforcing, and tracking caregivers’ use of skills). Although this study did not analyze engagement, other studies on group interventions with adults with depression have shown that AI could enhance the engagement with the treatment and improve outcomes [22]. For developers, including accessible resources (eg, apps) and voice features to avoid typing will be essential to ensure smoother engagement with the CAs. Finally, for health systems, integrating AI-supported interventions could lead to greater accessibility and cost efficiency, reducing logistical barriers, such as time, transportation, and provider shortages, that often limit families’ participation in evidence-based parenting programs.

### Limitations and Future Directions

Several limitations should be considered when interpreting the findings reported here. First, the study relied on real-world, cross-sectional, and self-report data, which may be influenced by recall or social desirability biases. Second, there was no control group or pre-/postmeasurement of child behavior change, preventing conclusions about child behavior outcomes or causal effects. Third, the sample likely reflects a degree of selection bias, as participating caregivers were digitally literate, motivated, and had consistent internet access, and we do not know the impressions of those who did not complete the surveys. Additionally, the percentages attributed to Pat and the live sessions represent subjective perceptions rather than objective estimates of causal contribution. Finally, because the study was conducted in Argentina and Paraguay, findings may be context bound and not fully generalizable to other cultural or health care settings. To note, most evidence-based parenting interventions (including PMT) have been developed and tested primarily in high-income, English-speaking contexts, often with non-Hispanic White samples [42]. This study provides a unique contribution to the field by including a Latin American population that often lacks mental health resources and is understudied.

Future research should build on these findings by using RCT designs to examine the causal effects of hybrid, AI-supported PMT compared to traditional formats. A key next step is to quantify the therapeutic alliance that may develop between caregivers and AI agents, given caregivers’ reports of empathy and support from Pat. Finally, future evaluations should incorporate child-level outcomes (eg, behavioral improvements) and family-level outcomes (eg, reductions in parenting stress and improvements in family functioning) to determine the broader impact of this approach on both caregivers and children.

### Conclusion

In this preliminary investigation, caregivers reported positive experiences with a hybrid program combining live videoconferencing group PMT sessions plus an AI. Delivering PMT in group format and through videoconferencing increases the scalability of the workforce and increases accessibility to the resources. Furthermore, the integration of AI (Pat) expanded the benefits of traditional PMT by continuing the delivery of skills and providing personalized, on-demand support between sessions. Caregivers valued Pat’s immediacy and practicality, noting that “Pat helps us with specific questions about how to talk to our child or how to handle situations,” as well as its constant availability, sharing that “having Pat 24/7 makes you feel supported at all times.” Together, these components created a synergistic model in which human expertise and empathy were complemented by AI-driven accessibility and continuity, reducing barriers, such as time, cost, and clinician availability. This hybrid approach appears to make PMT more flexible, scalable, and sustainable without sacrificing the relational quality that remains central to therapeutic success.

## Appendix

Multimedia Appendix 1 Graphical representation of the relative contribution of Pat or group sessions to the caregiver's child's progress.

## Abbreviations

AI: artificial intelligence
CA: conversational agent
IRR: interrater reliability
NPS: net promoter score
PMT: parent management training
RCT: randomized controlled trial

## Data Availability

The datasets generated or analyzed during this study are not publicly available due to difficulty in anonymizing qualitative messages but are available from the corresponding author upon reasonable request.
