# Supplementary figures and images for: Real-World Data From a Group Parent Management Training Program Enhanced Using Artificial Intelligence: Qualitative Study
Source: JMIR Form Res. 2026 Apr 7;10:e91841. doi: 10.2196/91841 (PMC13100577; doi:10.2196/91841)

## Appendix

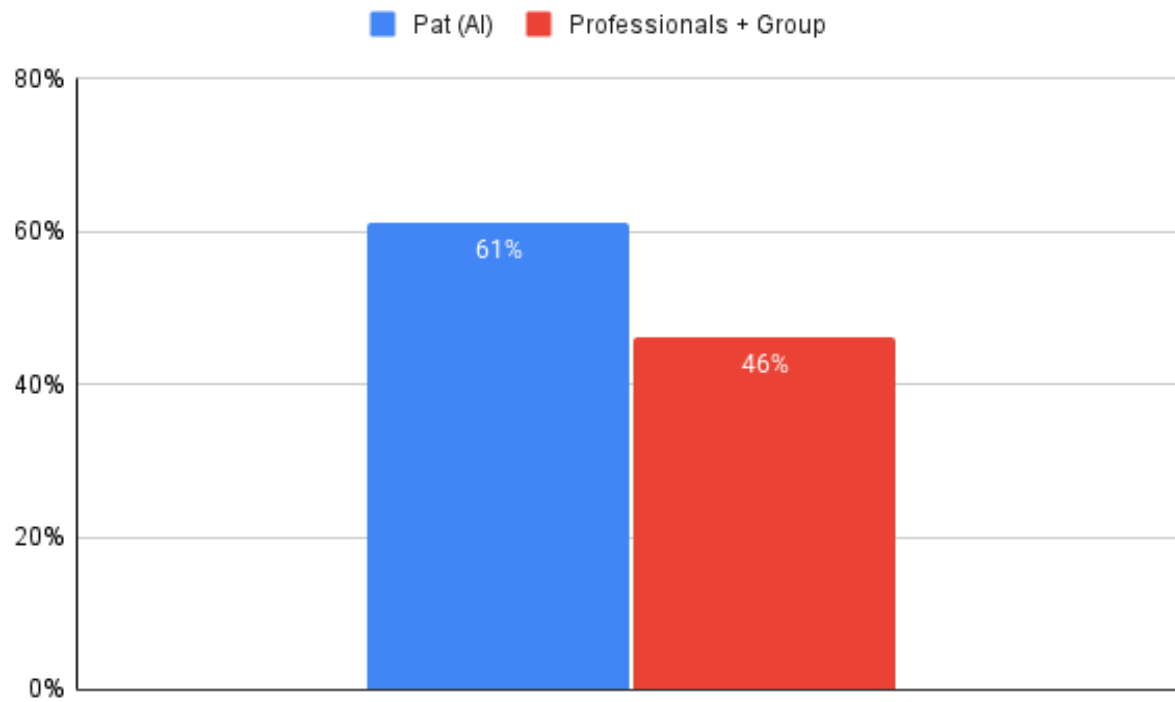

Relative contribution of Pat or group sessions to caregiver child's progress

Supplement: Multimedia Appendix 1 [file formative_v10i1e91841_app1.pdf]
